# Supplementary material for: The Evolution of Rag Gene Enhancers and Transcription Factor E and Id Proteins in the Adaptive Immune System
Source: Int J Mol Sci. 2021 May 31;22(11):5888. doi: 10.3390/ijms22115888 (PMC8199221; doi:10.3390/ijms22115888)
Supplement: Supplementary file 1 [file ijms-22-05888-s001.zip › ijms-1226271-supplementary.pdf]

Figure S1

(A)

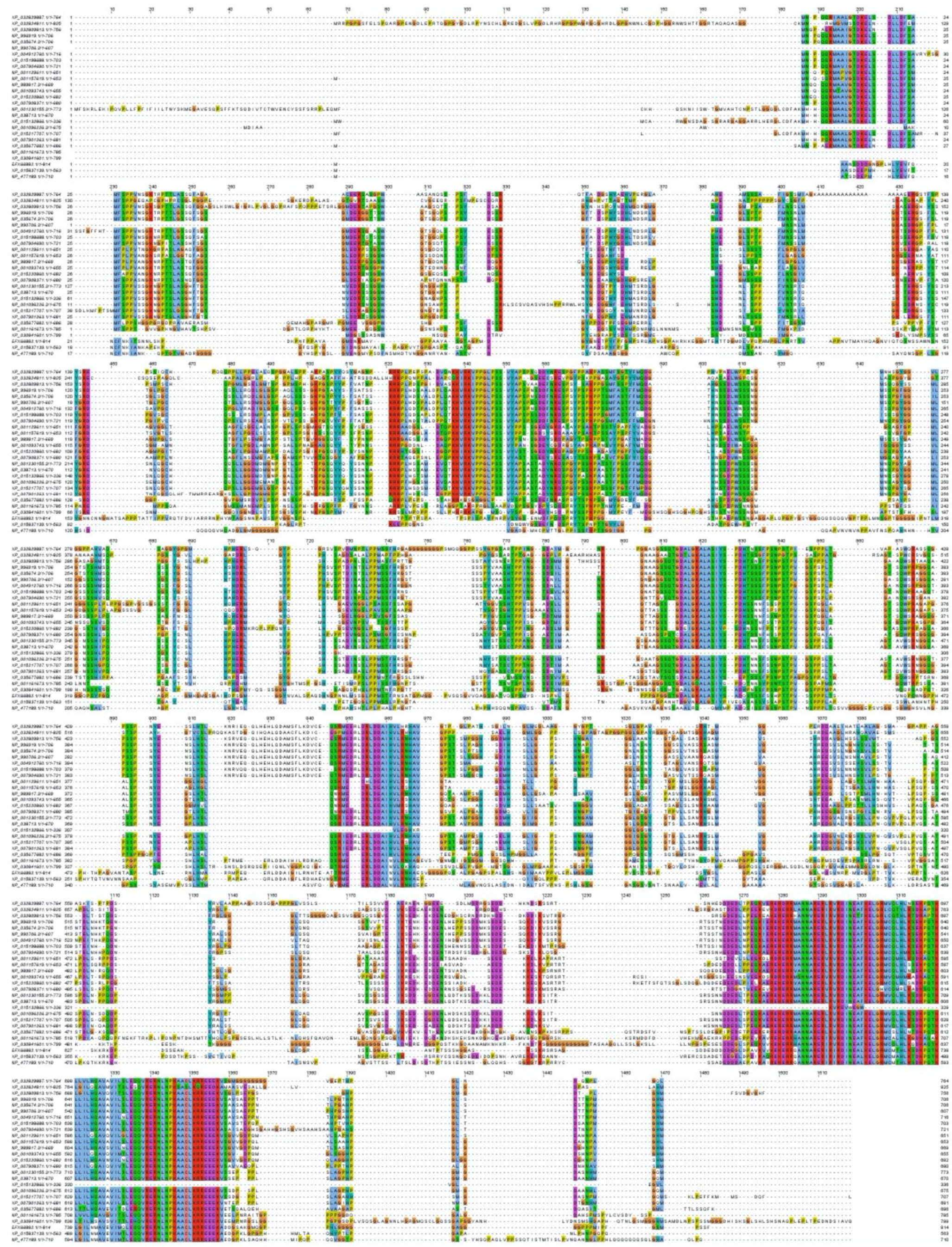

Figure S1

(B)

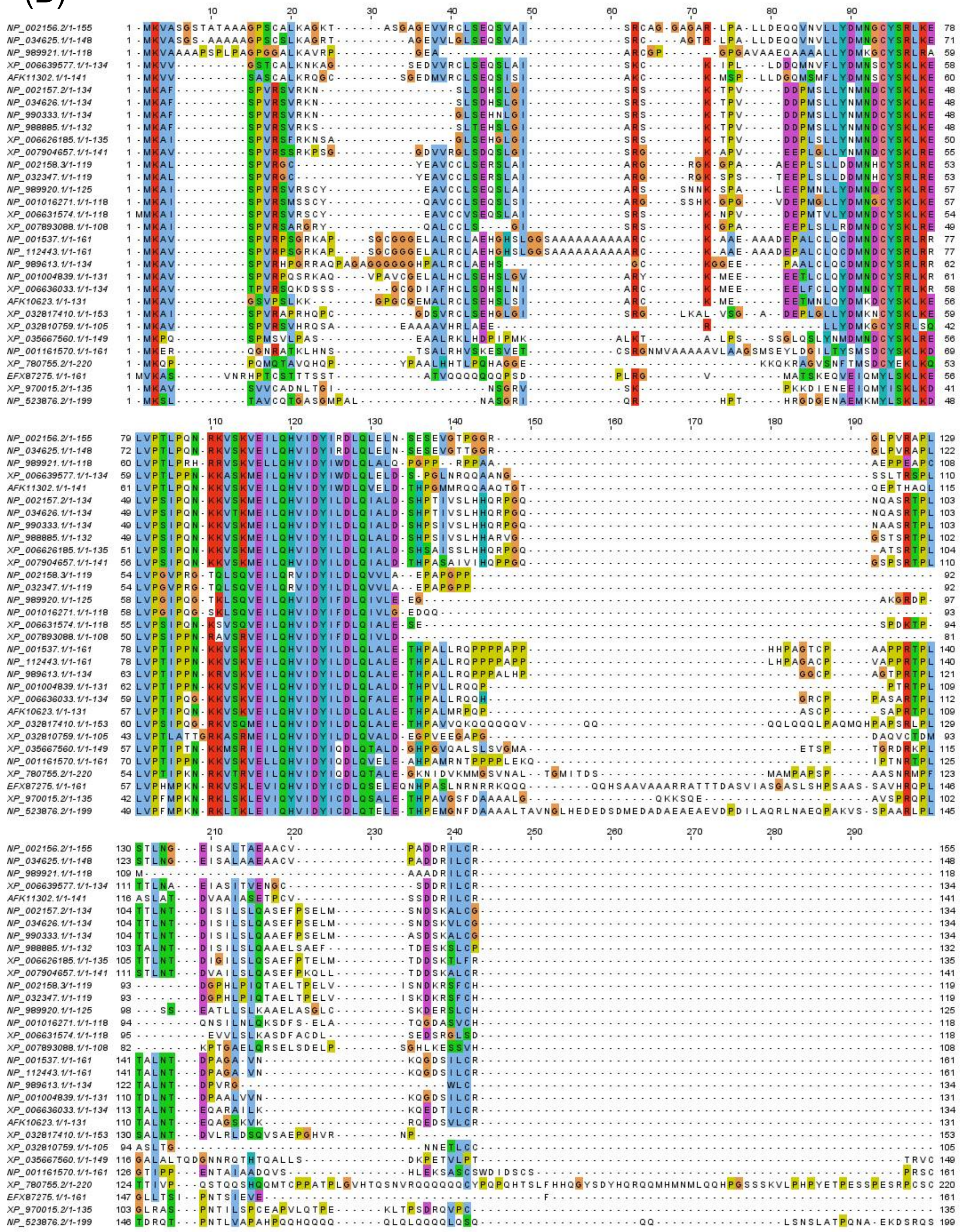

Figure S1. Multiple sequence alignments of E proteins (A) and Id proteins (B). The alignments were visualized using Jalview (version 2.11.1.4) [1].

1. Waterhouse, A. M.; Procter, J. B.; Martin, D. M.; Clamp, M.; Barton, G. J. Jalview Version 2 - a multiple sequence alignment editor and analysis workbench. Bioinformatics **2009**, 25(9), p. 1189-1191.
